# Waiting for pain: effect of a mindfulness intervention during a wait situation on pain intensity

**DOI:** 10.3389/fpain.2025.1653859

**Published:** 2026-01-14

**Authors:** Rebecca Stewing, Thomas Forkmann, Elisabeth Vögtle, Franziska Harms, Antonia Barke

**Affiliations:** 1 Department of Clinical Psychology and Psychotherapy, Faculty of Educational Sciences, Institute of Psychology, University of Duisburg-Essen, Essen, Germany; 2 Department of Clinical Psychology and Psychological Intervention, Faculty of Educational Sciences, Institute of Psychology, University of Duisburg-Essen, Essen, Germany; 3 Department of Clinical Psychology and Psychotherapy, Georg-Elias-Mueller Institute of Psychology, University of Goettingen, Goettingen, Germany

**Keywords:** acute pain, mindfulness, pain intensity, pressure pain, worry

## Abstract

**Background:**

Research has shown substantial evidence for the effectiveness of mindfulness-based interventions in the management of chronic pain. Less evidence is available whether a one-time mindfulness intervention may also be helpful in alleviating acute procedural pain. While anticipating a potentially painful procedure, people may worry about the upcoming pain. We investigated whether the time spent in a waiting room prior to an appointment could be used for a brief mindfulness intervention.

**Methods:**

The sample consisted of 93 female students. Experimental pain was induced with a pressure pain algometer. Subjective pain ratings were recorded with a numerical rating scale in a 3 × 2 mixed design with the factors condition (mindfulness, worry, control) and measurement time (baseline, post). A situation corresponding to a waiting room in primary care was created. Participants received an audio recording of a mindfulness meditation, a worry instruction, or an instruction to wait. In addition, participants answered the state version of the Mindful Attention Awareness Scale (State) at both measurement times.

**Results:**

3 × 2 mixed-design ANOVA showed no main effect for the within-subjects factor “measurement time” *F*(1, 89) = 0.11, *p* = .74, no main effect for the between-subjects factor “condition” *F*(2, 89) = .24; *p* = .98, and no interaction effect of “measurement time×condition” *F*(2, 89) = 2.53, *p* = .09. Analyses showed that worrying led to an increase in perceived pain intensity [*t*(31) = 1.74, *p* = .046 (one-tailed), *d* = 0.31]. No further effects were observed. State mindfulness in the mindfulness condition increased between the measurement times [*t*(29) = 2.00, *p* = .03 (one-tailed), *d* = 0.37].

**Discussion:**

Mindfulness increased through the induction; it did not affect perceived pain intensity. In the worry condition, perceived pain intensity increased, which is in accordance with research on detrimental effects of worry. Regarding the aim of the study, the experiment showed that a one-shot mindfulness intervention was able to promote state mindfulness, but not decrease perceived pain intensity. Future research should investigate whether mindfulness has more impact on the affective component of the pain, rather than on its sensory component.

## 1 Introduction

The majority of patients undergoing physical medical treatments, especially those with hospital admissions, experience acute procedural pain at least at one point in the course of their illness (1, 2) either due to their condition or as an accompaniment of medical procedures. Procedural pain is understood as pain as a result of a specific, limited, invasive procedure (3). The treatment of such pain is often inadequate (1, 2, 4) possibly due to the limited representation of the topic of acute pain in medical curricula (1, 4). Since pain is a biopsychosocial phenomenon, psychological interventions may be helpful options regarding procedural pain (5–9).

Psychological interventions are helpful in the management of chronic pain (1, 8–12). Cognitive behavioral therapy achieves small to medium positive effects on pain, disability, mood and catastrophizing (8, 9), and aims at a promotion of a realistic positive reappraisal. It implicitly assumes the presence of maladaptive behavior and the absence of adaptive behavior (e.g., concentration on pain vs. social interaction) (13). Mindfulness-based interventions may provide helpful alternatives to existing cognitive behavioral therapy methods. They have a good face-value for many people and can dispense with assumptions of maladaptive thoughts and behaviors. Mindfulness includes a non-judgmental attention to the moment-by-moment experience. It is characterized by an open and receptive attention (14). Mindfulness-based interventions are extensively used (9, 13, 15) and chronic pain patients reported significant positive changes regarding pain self-efficacy, pain acceptance, pain interference and catastrophizing (16, 17). Also, pain intensity ratings decreased in the course of mindfulness-based interventions (10). A brief body-mindfulness-intervention led to a significant reduction of pain-related distress and disability (18). If used in a helpful way, psychological interventions such as mindfulness-based interventions may even reduce the utilization of the healthcare system to a certain extent (19).

Less is known about benefits in acute procedural pain. Acute procedural pain accompanies many necessary diagnostic and curative interventions (1, 2). Here, psychological interventions also seem helpful. Pain tolerance significantly increased during a cold pressor test and distress was reduced after a mindfulness intervention (7). A three-day mindfulness intervention led to reduced ratings of pain intensity when confronted with electrical stimulation as well as decreased anxiety scores, rated with the State Anxiety Inventory (SAI) (20). Recent research comparing the effects of different mindfulness-based interventions with control or placebo conditions (21, 22) has shown a moderate effect on the pain intensity of mindfulness-meditation and loving-kindness meditation (22).

The intensity of pain is often exacerbated by worry regarding the pain and its consequences. Worry is defined as recurring fearful thoughts in relation to an impending event, often with the intention to prepare for it (23). Especially the expectation of impending pain is often associated with a high level of emotional stress (24), catastrophizing, worry and rumination (25–27), which increase the perceived intensity of pain and can make associated procedures and treatments more unpleasant (21, 22). A higher degree of catastrophizing was associated with higher subsequent pain intensity (16, 17, 26, 28). When designing medical waiting rooms, attempts are typically made to create a pleasant atmosphere and offer distraction in order to prevent such worry processes, for example by displaying magazines or hanging pictures (24). In general, psychological interventions such as mindfulness-based therapy may also reduce worry (16, 17, 25, 27–30). If mindfulness is practiced instead of worrying, the perceived pain intensity of an upcoming treatment may be reduced. However, since mindfulness is a mental stance that has to be practiced regularly (31), mostly people with respective training can fall back on mindfulness in anticipation of a painful procedure.

We wondered about the effect of different mental occupations while anticipating acute procedural pain. In particular, we were interested whether a brief, guided mindfulness meditation would reduce pain intensity in an experimental analogue of procedural pain even for people without prior meditation experience. In order to investigate this question, we created a waiting room situation in which the participants would either wait as usual and be allowed to read magazines or use their smartphone, or would be instructed to worry about the subsequent pain or receive a short-guided mindfulness meditation.

## 2 Material and methods

### 2.1 Ethics

The study was conducted according to the Declaration of Helsinki and approved by the internal review board of the Georg-Elias-Müller-Institute of Psychology, Göttingen, Germany. All participants received full information prior to the study and provided informed consent. The participants received money (8 €) or credit towards their coursework.

### 2.2 Experimental design

A 3 × 2 mixed design with the between-subject factor “condition” (mindfulness meditation, worry and control condition) and the within-subject factor “measurement time” (pre, post) was used. The participants were allocated randomly to the conditions and the experimenters were blind to the allocation.

Depending on the condition, participants either listened to a recording of a mindfulness meditation, a guided worry instruction, or simply an instruction to wait until the next instruction. All recordings were spoken by the same female mindfulness trainer. Each recording lasted 10 min and was delivered via headphones.

#### 2.2.1 Mindfulness meditation

In this condition, a breathing meditation was performed. This meditation is suitable for beginners (31) and was successful to increase mindfulness in previous studies (11, 32). The instruction of the mindfulness meditation by Liu and colleagues (7) was translated into German and adapted. The listeners were instructed to become aware of their breathing and to recognise arising thoughts and feelings without evaluating them.

#### 2.2.2 Worry

During the worry condition, the participants listened to a recording that asked them to focus on the impending pain. The description and the worries referred specifically to the pressure pain and the visual image of their finger under the pressure point of the algometer was evoked. The description was based on studies of rumination (5, 6). The recording had the same length and intonation as the mindfulness meditation.

#### 2.2.3 Control condition

The control group's recording instructed the participants that they should continue to wear the headphones and were allowed to occupy themselves freely within the next 10 min. Then the recording fell silent for 10 min. The situation of a waiting room during a doctor's visit was created, so that the participants had the opportunity to use their mobile phones, read typical waiting room magazines or simply wait. Only sleeping and leaving the room was not allowed. After about 10 min, a new instruction announced the continuation of the measurement.

### 2.3 Pain induction

**Figure 1:**
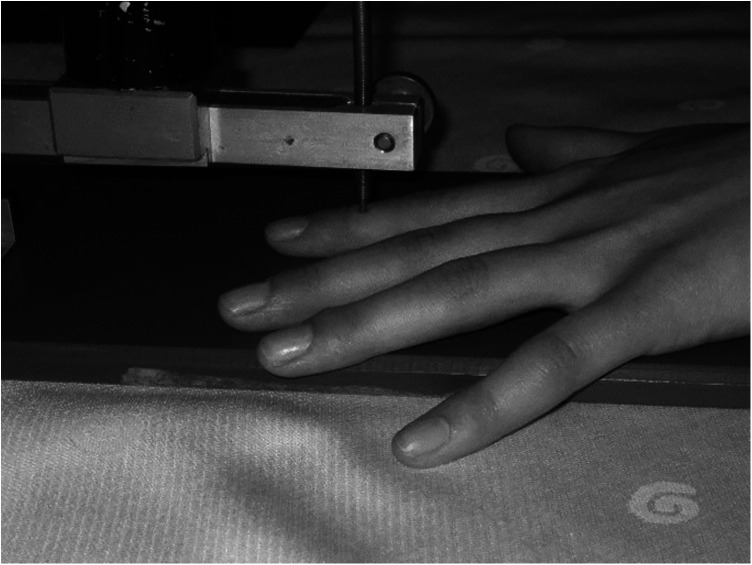
Pressure pain algometer.

Experimental pain was inducted with a custom-built pressure pain algometer, which applied a constant pressure of 0.92 MPa for 60 s on the middle phalanx of the selected fingers with a 3 mm^2^ blunt plunger (Figure 1). Each participant underwent two sets of three stimulations, one each on their forefinger, middle finger and ring finger. The order of the fingers was fully randomized.

### 2.4 Procedure

After they had provided informed consent, the participants answered state and trait questionnaires (see 2.5 Psychometric Measurements) on mindfulness and their current mood as well as on pain catastrophizing. In addition, the subjective assessment of mood, anxiety, worries and mindfulness was recorded using a visual analogue scale (VAS) from “not at all” to “extremely”. This was followed by the first pressure pain application (pre), which was framed as a calibration of the algometer. At the same time, pain intensity was assessed as a central outcome (see 2.5.1).

Subsequently, the participants were shown into a “waiting room” and asked to wear a set of headphones. In order to maintain the blinding of the experimenters, they received all instructions and the random allocation to one of the three conditions by headphone.

**Figure 2:**
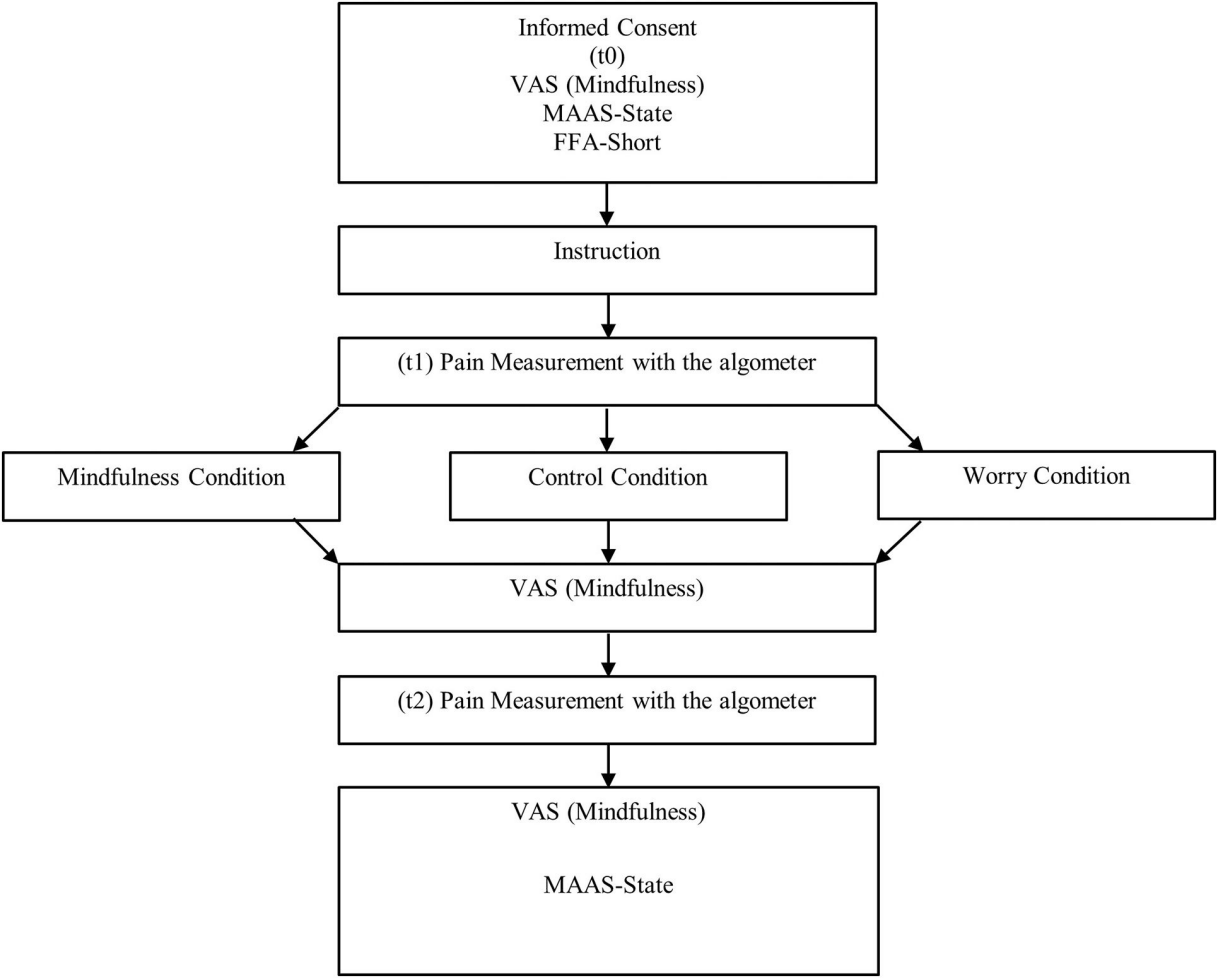
Procedure. VAS, visual analogue scale; MAAS-State, mindful attention awareness scale-state; FFA-Short, freiburg mindfulness questionnaire—short version.

At the end of the waiting-intervention phase, the pain measurement was repeated (post). After the second pain induction, the participants were asked how much they had worried since the beginning. The state questionnaires were presented again and basic information on age, gender, whether the participant had chronic pain, used any regular medication or had happened to use any on the day were recorded. In addition, the participants were asked whether they had previous experience with meditation and mindfulness practice and whether they had followed the instructions via the headphones (“I followed the instructions via the headphones”, 5-point Likert scale, “agree—disagree”). For a detailed overview of the procedure see Figure 2.

### 2.5 Psychometric measurements

#### 2.5.1 Pain intensity

During the pain application, the participants rated the pain intensity every 20 s on a numerical rating scale from 0 = “no pain” to 10 = “strongest pain imaginable”. For each set, the pain intensity was calculated as the mean value of the individual ratings after 20, 40 and 60 s across all three fingers. This resulted in one value per participant before the intervention (pre) and one value after the intervention (post).

#### 2.5.2 Mindfulness

##### 2.5.2.1 Single-item visual analogue scales

We used visual analogue scales to measure the subjective assessment of mindfulness at several time points (“Please mark on this line with a vertical line how much you feel in the here and now”). The line measured 100 mm and the left end of the VAS was marked with “not at all in the here and now” the right end with “completely in the here and now”. The VAS mark was converted into values of 0–100 with higher values signifying a higher degree of mindfulness. The single-item VAS was used prior to the second pain induction to assess whether the experimental manipulation had any effect without using too long a time or too many items that may disrupt any mindfulness state attained.

##### 2.5.2.2 Mindful Attention Awareness Scale - State Version (MAAS-State)

Mindful Attention Awareness Scale - State Version (MAAS-State) (33) was used to record state mindfulness before and after the experiment. The state version was extracted from the long version of Brown and Ryan by Brown and Ryan themselves (33). As only the long version exists in German (α = .83) (34, 35), we used the corresponding items of the English MAAS-State version (α = .92) (33) for this study. The questionnaire includes five items with negative wording (e.g., “I was finding it difficult to stay focused on what was happening”.) and is to be answered on a seven-point Likert scale (0 = “not at all”; 6 = “very much”). The items referred to the current moment (“Please answer as you really experience these things at the moment…”). Higher values in the questionnaire represent a less mindful state. Before the analysis, the values were inverted so that higher values indicate higher mindfulness. Mean scores per participant are reported. The internal consistencies for the present sample were α_pre_ = .76 at the first measurement point and α_post_ = .73 at the second measurement point.

##### 2.5.2.3 Freiburg Mindfulness Questionnaire - short version (FFA)

The Freiburg Mindfulness Questionnaire (Freiburger Fragebogen zur Achtsamkeit) was used in its short version (36). The questionnaire is used to assess mindfulness as a trait (37). The short version includes 14 items (e.g., “I am open to the experience of the moment.”; “I am impatient with myself and my fellow human beings”). The participants were asked to rate the items on a 4-point Likert scale from 0 = “almost never” to 3 = “almost always”. Mean scores per participant are reported. The internal consistency for the present sample was α = .68.

#### 2.5.3 Worries

We assessed how much worries the participants had experienced at the end of the experiment (“How worried have you been since with regard to the upcoming pain application, from 0 not all to 100 extremely worried”). The single-item assessment was used to ascertain whether the experimental manipulation had any effect on state worry.

### 2.6 Participants

An *a priori* power analysis using G*power (38) was calculated. As a three-day mindfulness training programme showed a large effect (20), we assumed a small effect for our brief mindfulness intervention programme. Based on an effect size of *f* = 0.15 with a power of *β* = 0.80 (*α* = 0.05), it was determined that a minimum number of *n* = 111 participants was necessary to find an interaction when using a 3 (mindfulness condition, control condition, worry condition) × 2 (pre, post) ANOVA.

The participants were recruited with postings at the Georg-Elias-Müller Institute Göttingen, Germany, via social media as well as through advertising in psychology lectures. Since pain reports may be affected by the gender of the experimenter (39, 40), it was decided to avoid interaction of experimenter and participant gender by examining only women by two female experimenters. Exclusion criteria were: previous meditation experience and diabetes. People with meditation experience were excluded, as meditation experience may interact differentially with the experimental conditions. Persons with diabetes were excluded in order to prevent any risk of vascular injury from the pressure during the application of pressure pain (41). 114 participants took part in the study. Of these, 21 stated that they already had experience with meditation and were excluded from the further investigation. The final sample consisted of 97 participants with an age range from 18 to 40 years (*M* = 22.9; *SD* = 3.6).

### 2.7 Data analysis

The effects of the experimental conditions and measurement time were investigated with a mixed 3 × 2 ANOVA with the factors condition (Mindfulness, Worry, Control) and measurement time (pre, post).

To assess the success of the experimental manipulation regarding mindfulness, pre-post differences in state mindfulness were calculated in all groups by paired one-tailed *t*-tests.

A Single-factor ANOVA were used to analyze the differences in the degree of worry between the conditions after the second measurement and follow-up t-tests were used to investigate the differences in more detail.

Subsequent exploratory analyses were conducted to gain a better understanding of the effect of worry and mindfulness on pain intensity by comparing the pain intensity before and after the intervention with paired *t*-tests within the mindfulness and the worry group.

## 3 Results

### 3.1 Manipulation success

A 3 × 2 mixed-design ANOVA (condition × measurement time) for mindfulness showed a main effect for the factor “measurement time”, no main effect for the factor “condition” and no interaction effect “condition × measurement” time (see Table 1 for means and standard deviations and Table 2 for the results of the ANOVA).

**Table 1:** Results of the 3 × 2 analysis of variance with the dependent variable mindfulness (mindful attention awareness scale-state; MAAS); *n* = 93.

Effect	*df*	Mean of squares	*F*	*p*	*η*_p_^2^
Condition	2	1.44	0.97	.38	0.021
Measurement Time	1	0.95	4.54	.04	0.048
Condition × Measurement Time	2	0.59	2.83	.06	0.059

**Table 2:** Mean values and standard deviations for the whole sample and each condition.

Variables	All (*n* = 93)		Mindfulness (*n* = 30)		Worry (*n* = 33)		Control (*n* = 30)	
	Mean	SD	Mean	SD	Mean	SD	Mean	SD
Age (years)	22.9	3.6	23.0	3.07	22.9	4.54	22.7	2.93
Trait mindfulness (FFA Short; 0–3)	2.56	0.77	2.73	0.35	2.78	0.36	2.67	0.26
Pain intensity pre (VAS 0–10)	4.16	1.69	4.24	1.62	4.05	1.71	4.19	1.79
Pain intensity post (VAS 0–10)	4.10	1.59	4.10	1.70	4.23	1.69	3.98	1.44
State mindfulness pre (MAAS State; 0–6)	3.83	0.95	3.84	1.05	3.78	0.84	3.89	0.98
State mindfulness post (MAAS State; 0–6)	3.97	0.89	4.10	0.89	3.70	0.75	4.14	0.99
Worries post (VAS 0–100)	21.77	20.02	16.6	18.3	30.0	21.6	18.1	17.6

**Figure 3:**
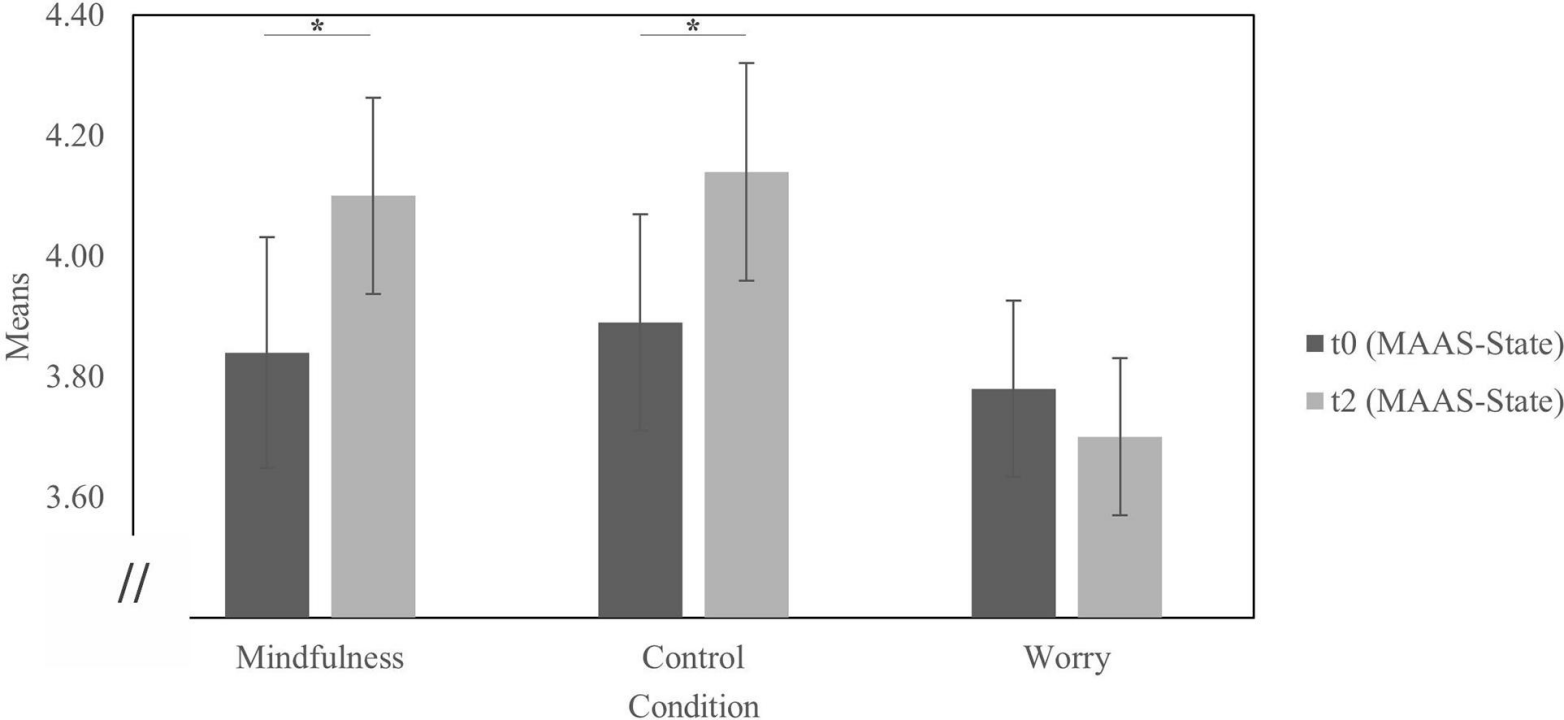
Mean values of the mindful attention awareness scale—state (MAAS-state). *N* = 93; pre: time point before first pain measurement; post: time point after second pain measurement; mindfulness: *n* = 30, *M_pre_* = 3.84, *SD_pre_* = 1.05, *M_post_* = 4.10, *SD_post_* = 0.89; control: *n* = 30, *M_pre_* = 3.89, *SD_pre_* = 0.98, *M_post_* = 4.14, *SD_post_* = 0.99; worry: *n* = 33, *M_pre_* = 3.78, *SD_pre_* = 0.84, *M_post_* = 3.70, *SD_post_* = 0.75; *p* < .05.

In the mindfulness condition, the participants' mindfulness increased from baseline to the post measurement [*t*(29) = 2.00, *p* = .03 (one-tailed), *d* = 0.37], as it did in the control group [*t*(29) = 1.80, *p* = .04 (one-tailed), *d* = 0.33], but not in the worry group [*t*(32) = 1.02, *p* = .16 (one-tailed), *d* = 0.18] (Figure 3 for details).

A single-factor ANOVA showed that the groups' worries differed after the waiting room intervention [*F*(2, 89) = 4.55, *p* = .01; *η*^2^ = 0.09]. The participants in the worry condition worried more than the participants in the mindfulness [*t*(60) = 2.63, *p* = .005 (one-tailed), *d* = 0.67] and the control conditions [*t*(60) = 2.37, *p* = .01 (one-tailed), *d* = 0.60]. The mindfulness and control conditions did not differ from each other in terms of worry [*t*(58) = 0.33, *p* = .37 (one-tailed), *d* = 0.07].

### 3.2 Main analysis

The 3 × 2 mixed-design ANOVA (condition × measurement time) for pain intensity showed no main effects and no interaction “condition × measurement time” (Table 3).

**Table 3:** Analysis of variance: pain intensity; *n* = 93.

Effect	*df*	Mean of squares	*F*	*p*	*η*_p_^2^
Condition	2	0.12	0.24	.98	0.001
Measurement Time	1	0.05	0.11	.74	0.001
Condition × Measurement Time	2	1.02	2.53	.09	0.054

### 3.3 Exploratory analyses

**Figure 4:**
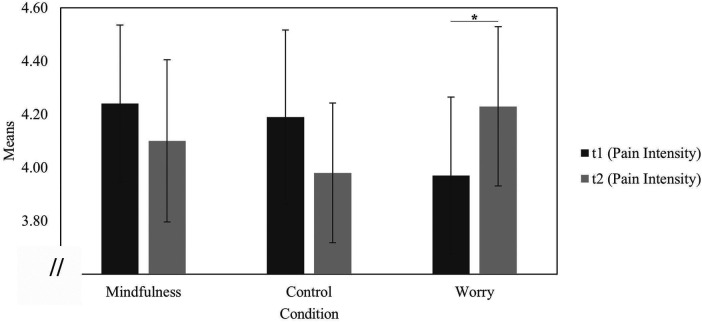
Mean values of the pain intensity ratings. *N* = 93; pre first pain measurement; post: second pain measurement; mindfulness: *n* = 30, *M_pre_* = 4.24, *SD_pre_* = 1.62, *M_post_* = 4.10, *SD_post_* = 1.67; control: *n* = 30, *M_pre_* = 4.19, *SD_pre_* = 1.79, *M_post_* = 3.98, *SD_post_* = 1.44; worry: *n* = 32, *M_pre_* = 3.97, *SD_pre_* = 1.67, *M_post_* = 4.23, *SD_post_* = 1.69; *p* < .05.

Within the mindfulness group, pain intensity before and after the intervention was compared with a paired *t*-test and showed no significant decrease in perceived pain intensity [*t*(29) = 0.88, *p* = .19 (one-tailed), *d* = 0.16]. The parallel comparison within the worry group revealed increased pain intensity [*t*(31) = 1.74, *p* = .046 (one-tailed), *d* = 0.31) (Figure 4).

## 4 Discussion

The present study investigated the effect of different mental occupations while anticipating acute procedural pain. The omnibus test showed no interaction effect for condition and measurement time. Detailed investigation revealed that a brief guided mindfulness meditation did not reduce ratings of pain intensity, but a brief worry instruction increased them.

The randomization was successful: At baseline, the conditions did not differ regarding mindfulness. After the brief intervention, mindfulness increased in the mindfulness condition and worry increased in the worry condition. Despite the absence of an intervention in the control group, mindfulness also increased during the waiting period.

### 4.1 Mindfulness condition

Although the manipulation increased mindfulness, the increased mindfulness did not reduce the perceived pain intensity. These results are inconsistent with the findings of previous studies which showed an effect of mindfulness on pain intensity of experimental pain stimuli (20, 42). Furthermore, mindfulness was able to increase pain tolerance and reduce distress during a cold pressure task (7).

A difference between our intervention and those of previous studies is the length of the training. Mindfulness is generally considered to be a practices method (31, 43). In the present study, participants were played a 10 min audio recording. Although this led to higher mindfulness scores, it is unclear whether participants could maintain this state of mindfulness during the pain induction. Previous studies have investigated the effect of mindfulness with training lengths ranging from 45 min to about 2.5 h a day over 10 weeks (10, 16, 20, 31). While the induction dose in the present study was clearly sufficient to increase self-reported mindfulness, it may have been not sufficient to reduce pain intensity. It could be speculated that maintaining a mindful state in the absence of adverse stimuli may be easier and thus require less practice than doing so in the presence of acute pain. It is possible that structured long-term training is required here, which focuses on the participants being able to apply the technique in situations that are normally detrimental to mindful acceptance. Unfortunately, mindfulness was not measured again after the pain application, so that this remains speculative. Especially in the area of chronic pain, longer programs with several sessions are offered, in which the perception of pain and how to deal with it, as well as mindfulness, are repeatedly monitored (44). In addition, there were variations in the pain modalities used in the different studies. Electrical stimulation, cold and heat stimulation and cold pressure task were used to induce pain (7, 20, 42). This can cause difficulties in the comparability of the individual results across different studies. Furthermore, many studies did not blind the investigators (7, 20, 38, 42), which was taken into account in the present study. It was shown that better assumptions by patients and investigators about the treatment of back pain led to higher treatment effects (45). This suggests that proper blinding tends to result in smaller effects of interventions.

### 4.2 Worry condition

Inducing worry led to increased self-reported worry and heightened pain intensity. Participants in the worry condition were significantly more worried after the instruction than participants in the other conditions. Worry induction could lead to high catastrophizing and anxiety, which in turn negatively influences the perception of pain. The associations between worry and anxiety and an increased pain intensity are well known (26, 27, 46, 47). Similar results in relation to pain catastrophizing in the context of acute pain were also shown by Cimpean and David (48). Here, pain catastrophizing under aversive conditions predicted lower pain tolerance in the cold pressure test and increased pain-related anxiety compared to neutral conditions.

Jensen and colleagues tried to explain this with a two-factor model that employs Gray's model of emotion regulation. The model postulates a behavioural activation system (BAS), which includes the cognitive content, that pain is not directly harmful and thus creates hope (emotion) and a sense of purpose (behaviour).The second system is a behavioural inhibition system (BIS), which sees pain as harmful (cognitive content) and thus leads to fear (emotion) and avoidance (behaviour) (49), these results could be explained by an activation of the BIS, which leads to a higher sensitivity to pain. According to Jensen's and colleagues' (49) explanation, one might speculate that the cognitive content (pain as harmful) was activated with the worry recording.

### 4.3 Control condition

The participants of the control group, who were simply instructed to wait and pass the time as they might in a doctor's waiting room, e.g., by perusing the magazines provided or quietly using their mobile phone, also reported increased mindfulness. One might speculate about two possible explanations: Firstly, the increase could be the result of straightforward relaxation. In both conditions, the mindfulness condition and the control condition, the participants were forced to sit quietly without being disturbed for 10 min. Even the opportunity of reading some magazine might take away their mind from the bustle of their normal day. In the worry condition, such relaxation may have been prevented by the worry instruction.

Secondly, Senker et al. (50) showed that regularly asking about mindfulness may promote a mindful attitude. In our study, participants were asked several times within 20 min about their mindfulness, thereby possibly increasing their mindfulness. In the worry condition, the participants were asked the same number of times, but it seems plausible that the worry audio counteracted any mindfulness that might have been induced by the repeated questions.

### 4.4 Validity

Internal validity was ensured by completely blinding the investigators and the participants. Neither the investigators knew which audios the participants were listening to, nor were the participants aware of the purpose and objectives during the survey. Instead, they were informed about them afterwards. The standardization of the intervention and waiting condition was ensured by the same length and the same person speaking in the same tone. The procedure was also standardized. In addition, baseline checks were carried out before the first induction of a pain stimulus.

The standardized laboratory setting reduced the external validity, however. In the present study, acute pain stimuli were used in a controlled experimental setting from which the participant could leave at any time. This differs in crucial ways from pain occurring in everyday life (51, 52): Firstly, the participants in the present study made an informed and voluntary decision to take part in the study and to undergo pain induction. Secondly, the participants knew the cause and source of the pain. Thirdly, they knew the pain was harmless, and finally, they could choose to withdraw at any moment. The experience of control and self-efficacy can significantly impact pain perception (12, 53, 54).

Generally, pain is a multidimensional concept and encompasses both sensory and affective components (21). It is well known that pain involves cortical activations, in areas related to sensory as well as affective aspects (55). A study of patients with pain-sensitive teeth in a simulation of painful dental treatment shows activation not only in the sensory-discriminative areas, but also in the emotional-cognitive regions (56). In the present study, only the sensory pain component was assessed. It is possible that—rather than pain intensity—pain unpleasantness is a more appropriate target for mindfulness. Positive effects of mindfulness on pain unpleasantness have been shown (21, 22). Here, participants with chronic pain were asked to rate the unpleasantness and intensity of their pain and then received either a recorded 20-minute mindfulness meditation, a loving-kindness mediation or an audiobook in the control condition, each of the same length (22). In the other study, participants also received a 20-minute focussed attention mindfulness, a specific sham mindfulness or a general sham mindfulness (21).

### 4.5 Limitations

The results of the present study have to be appreciated in the light of its strengths and limitations. A strength of the study is its thorough methodology with blinded experimenters, clear exclusion criteria (e.g., people with meditation experience), and the randomized experimental design. The present study is the first to use an experimental design to create a waiting room situation and investigate the effects of worries and mindfulness on pain intensity of subsequent acute pain. The study was adequately powered for small effects. Our analyses showed that both our randomization and manipulation for mindfulness were successful. Limitations were the exclusive focus on the sensory aspect of pain and repetitive questions about the participants' mindfulness. Due to the focus on a healthy student sample, chronic pain or pain medication was not assessed. Furthermore, the inclusion of only female participants limits the generalisability of the results, as biological sex influence pain perception (57).

### 4.6 Implications

The present findings underline the importance of adequate strategies for dealing with the expectation of acute pain in medical settings, in particular preventing patients from worrying about the incumbent procedures. Future studies should investigate which aspect of acute pain would be the most promising target of psychological strategies such as mindfulness. In this context, it is also interesting to consider the impact these strategies may have on anticipatory anxiety.

## Data Availability

The raw data supporting the conclusions of this article will be made available by the authors, without undue reservation.
